# A Novel Iron Transporter SPD_1590 in *Streptococcus pneumoniae* Contributing to Bacterial Virulence Properties

**DOI:** 10.3389/fmicb.2018.01624

**Published:** 2018-07-20

**Authors:** Xinyu Miao, Jiaojiao He, Liang Zhang, Xinlu Zhao, Ruiguang Ge, Qing-Yu He, Xuesong Sun

**Affiliations:** ^1^Key Laboratory of Functional Protein Research of Guangdong Higher Education Institutes, Institute of Life and Health Engineering, College of Life Science and Technology, Jinan University, Guangzhou, China; ^2^Key Laboratory of Gene Engineering of the Ministry of Education, State Key Laboratory of Biocontrol, College of Life Sciences, Sun Yat-sen University, Guangzhou, China

**Keywords:** iron transporter, *S. pneumoniae*, proteomics, bacterial virulence, hemin binding

## Abstract

*Streptococcus pneumoniae*, a Gram-positive human pathogen, has evolved three main transporters for iron acquisition from the host: PiaABC, PiuABC, and PitABC. Our previous study had shown that the mRNA and protein levels of SPD_1590 are significantly upregulated in the Δ*piuA*/Δ*piaA*/Δ*pitA* triple mutant, suggesting that SPD_1590 might be a novel iron transporter in *S. pneumoniae*. In the present study, using *spd1590*-knockout, -complemented, and -overexpressing strains and the purified SPD_1590 protein, we show that SPD_1590 can bind hemin, probably supplementing the function of PiuABC, to provide the iron necessary for the bacterium. Furthermore, the results of iTRAQ quantitative proteomics and cell-infection studies demonstrate that, similarly to other metal-ion uptake proteins, SPD_1590 is important for bacterial virulence properties. Overall, these results provide a better understanding of the biology of this clinically important bacterium.

## Introduction

Metal ions play a vital role in the survival and virulence of bacteria (Faulkner and Helmann, 2011). Iron is an essential transition metal for most living organisms, as it is involved in redox reactions and functions as a cofactor of many proteins, such as cytochromes, ferredoxins, and other iron-sulfur proteins (Chua et al., 2007; Ratledge, 2007). In host cells, iron is mostly stored as hemoglobin, and its availability is limited by the host as a defense mechanism against invading pathogens (Turner et al., 2017).

*Streptococcus pneumoniae* is a major Gram-positive human pathogen. It has the capacity to colonize the mucosal surface of the upper respiratory tract and can cause a range of diseases, including pneumonia, otitis media, and meningitis, particularly in immunocompromised individuals (Kadioglu et al., 2008). In response to iron limitation by the host, *S. pneumoniae* has evolved multiple efficient iron-uptake strategies, including the use of ATP-binding cassette (ABC) transporters (Rees et al., 2009; Seeger and Van Veen, 2009). The three major iron-ABC transporters in *S. pneumoniae* are PiaABC, PiuABC, and PitABC, which transport ferrichrome, hemin, and ferric ion, respectively (Brown et al., 2001, 2002; Whalan et al., 2006).

In a previous study, we showed that a Δ*piuA/*Δ*piaA/*Δ*pitA* triple mutant is able to grow, albeit slowly, in an iron-containing medium (Yang et al., 2016), which suggests that there are likely to be additional iron-uptake systems in *S. pneumoniae*. In that work, using translatomics and proteomics, we identified some differentially expressed proteins in the triple mutant strain compared to the D39 wild-type (WT) strain. Notably, the expression of *spd1590*, of unknown function, was upregulated 66.9-fold at the mRNA level and 3.49-fold at the protein level.

The aim of the present study was to explore the biological function of SPD_1590. To this end, *spd1590*-knockout, -complemented, and -overexpressing strains were constructed. *In vitro* and *in vivo* experiments were used to characterize the biochemical properties and the contribution to bacterial virulence properties of SPD_1590. This study provides insight for a better understanding of iron transportation in *Streptococcus*.

## Materials and Methods

### Sequence Analysis

The protein sequence of SPD_1590 from *S. pneumoniae* D39 was used as a seed to query the NCBI database using the Protein BLAST tool. Then, multiple sequence alignment and cluster analysis were performed with high-scoring proteins using the software package Clustal-X 2.1.

### Growth Media and Culture Conditions of Bacterial Strains

*Streptococcus pneumoniae* D39 was cultured in THY medium, composed of Todd-Hewitt broth (Oxoid, United Kingdom) supplemented with 0.5% yeast extract (Oxoid, United Kingdom), or grown on Columbia Agar (Difco, United States) containing 5% sheep blood (Ruite, China) at 37°C in an incubator containing 5% CO_2_. *Escherichia coli* BL21 and DH5α were cultured in Luria-Bertani (LB) medium at 37°C in a shaking incubator. To select positive colonies, erythromycin (Erm; Sigma, United States) at 0.25 μg/ml, chloramphenicol (Cm; Sigma, United States) at 4 μg/ml, or ampicillin (Amp; Sigma, United States) at 100 μg/ml was added to the medium.

The iron-restricted medium was prepared by adding 5% Chelex-100 resin (Bio-Rad, United States) to THY for 8 h with continuous agitation, followed by filter sterilization to remove the resin and supplemented with 100 μM CaCl_2_ and 2 mM MgCl_2_. When necessary, an iron source like hemin or ferric iron was added to the medium.

To determine bacterial growth curves, the C+Y medium was used (Lacks and Hotchkiss, 1960). This chemically defined medium contained the following ingredients per liter: 5 g casein hydrolysate (vitamin-free casamino acids, Difco, United States), 6 mg tryptophan, 35 mg cystine, 2 g sodium acetate, 8.5 g K_2_HPO_4_, 0.5 g MgC1_2_⋅6H_2_O, 2.5 mg CaC1_2_, 25 μg MnSO_4_⋅4H_2_O, 0.5 μg FeSO_4_⋅7H_2_O, 0.5 μg CuSO_4_⋅5H_2_O, 0.5 μg ZnSO_4_⋅7H_2_O, 0.2 μg biotin, 0.2 mg nicotinic acid, 0.2 mg pyridoxine-HC1, 0.2 mg thiamine-HC1, 0.1 mg riboflavin, 0.6 mg calcium pantothenate, 2 g glucose, 0.48 g bovine serum albumin, and 5 g yeast extract. All the bacterial strains and plasmids used were listed in **Table 1**.

**Table 1:** Bacterial strains and plasmids used in this study.

Strain or plasmid	Relevant characteristic(s)	Source or reference
***S. pneumoniae* strains**		
D39 (WT)	Wild-type	American type culture collection (ATCC, United States)
Δ*spd1590*	In-frame *spd1590* mutant strain derived from D39; Erm^r^	This study
Δ*piuA*/Δ*piaA*/Δ*pitA*	In-frame *piuA*/*piaA*/*pitA* mutant strain derived from D39; Erm^r^Cm^r^Spec^r^	Yang et al., 2016
Δ*spd1590*/pIB169-*spd1590*	Δ*spd1590* strain transformed with pIB169-*spd1590*; Erm^r^ Cm^r^	This study
D39/pIB169-*spd1590*	D39 strain transformed with pIB169-*spd1590*; Cm^r^	This study
***E. coli* strains**		
BL21	Wild-type	Invitrogen (United States)
BL21/pBAD-His*-spd1590*	BL21 strain transformed with pBAD-His*-spd1590*; Amp^r^	This study
**Plasmids**		
pBAD/HisA	pBAD/HisA vector contained *araBad* promoter; Amp^r^	Invitrogen (United States)
pBAD/HisA-*spd1590*	*S. pneumoniae* D39 *spd1590* fragment cloned into pBAD/HisA; Amp^r^	This study
pIB169	Shuttle plasmid contained P*_veg_* promoter; Cm^r^	Biswas et al., 2008
pIB169-*spd1590*	*S. pneumoniae* D39 *spd1590* fragment cloned into pIB169; Cm^r^	This study

*Erm^r^, erythromycin resistance; Cm^r^, chloramphenicol resistance; Spec^r^, spectinomycin resistance; Amp^r^, ampicillin resistance.*

### Construction of Mutant Strains

Using flanking homology polymerase chain reaction (LFH-PCR) (Wach, 1996) and the primers listed in **Table 2** (1590-P1 to pIB169-*spd1590*-R), the target gene *spd1590* was replaced with an antibiotic resistance cassette gene (*erm*) to construct Δ*1590*. The product of LFH-PCR, containing the upstream (621 bp) and downstream (530 bp) sequences of *spd1590* and the *erm* gene (860 bp), was incubated with the WT D39 strain stimulated with CSP_1_ (10 ng/ml) at 37°C for 2 h. Then, the transformants were transferred to an Erm-containing Columbia sheep blood agar plate for selection of positive strains, in which *spd1590* had been completely replaced by *erm*. The Δ*1590* strain was stocked after seven to eight sequential passages in THY medium with 0.25 μg/ml Erm to stabilize bacterial resistance. For construction of *spd1590*-overexpressing and -complemented strains, the *spd1590* gene was inserted into the pIB169 plasmid, containing an *amp* resistance cassette, to construct the pIB169-*spd1590* plasmid using the ClonExpress II One Step Cloning Kit (Vazyme, China), and the recombinant plasmid was transferred into *E. coli* DH5α for amplification. The pIB169-*spd1590* plasmid was extracted from *E. coli* DH5α and then transferred to the WT D39 and Δ*1590* strains, respectively, which had been stimulated with CSP_1_. Transformants were selected on Amp-containing Columbia sheep blood agar plates. All mutant strains were further confirmed by western blotting.

**Table 2:** The primers used in this study.

Primers	Sequence (5′–3′)
1590-P1	CTTGACAATTTTCCTTCC
1590-P2	AGGGGAGATAGAAAGCAAAC
1590-P3	ATCAAACAAATTTTGGGCCCGGCAACTCCCAGTGCT
	CCTA
1590-P4	ATTCTATGAGTCGCTGCCGACTGGTGATGAAAAAATG
	GAAAA
1590-F	AAATCATTGGTCTTTCAC
1590-R	GCTTCTACACCTTCGCTA
*erm*-F	AGTCGGCAGCGACTCATAGAAT
*erm*-R	CCGGGCCCAAAATTTGTTTGAT
pIB169-*spd1590*-F	GGAGACCGCGGTCCCGAATTCATGAAATTTAGAAAATT
	AGCTTGTACAGTAC
pIB169-*spd1590*-R	GGTCGACCTCGAGGGATCCGTGATGGTGAT
	GGTGATGTTGTTTCATAGCTTTTTTGATTG
CCL2-F	CAGCCAGATGCAATCAATGCC
CCL2-R	TGGAATCCTGAACCCACTTCT
IL-1β-F	AGCTACGAATCTCCGACCAC
IL-1β-R	CGTTATCCCATGTGTCGAAGAA
IL-6-F	ACTCACCTCTTCAGAACGAATTG
IL-6-R	CCATCTTTGGAAGGTTCAGGTTG
β-Actin-F	ACGTGGACATCCGCAAAG
β-Actin-R	GACTCGTCATACTCCTGCTTG
16S rRNA-F	CTGCGTTGTATTAGCTAGTTGGTG
16S rRNA-R	TCCGTCCATTGCCGAAGATTC
pspA-F	ATCTCCCGTAGCCAGTCAGT
pspA-R	GAGCAGCTTTTGCATCATCT
cbpD-F	CATTACTCAAACGAAAGGCTACAAA
cbpD-R	AGCAGCTGGTGATAGTGTCCATGCC
lytA-F	CTGTTGATAATGGTGCCT
lytA-R	TCATCTGCTAGATTGCGT
nanA-F	GAAATCGCAGAGTATAAGG
nanA-R	GTAAACAGACCAAGGAAGA

### Growth Curve Analysis

The WT and mutant strains were inoculated into C+Y medium at equal inoculation doses at 37°C in a 5% CO_2_ incubator. The optical density at 600 nm (OD_600_) was continuously measured every hour by UV-visible spectroscopy (Evolution 300, Thermo Fisher Scientific, United States) for 12 h. All data were analyzed by GraphPad Prism 6.0 (GraphPad Software, United States).

### Cloning, Expression, and Purification of SPD_1590

The target gene *spd1590* was cloned from *S. pneumoniae* D39 genomic DNA by PCR. The PCR product and pBAD/HisA plasmid were digested with restriction enzymes *Xho*I and *Kpn*I (TaKaRa, Japan), respectively. Next, the fragments were ligated with the ClonExpress II One Step Cloning Kit (Vazyme, China) to construct the fusion plasmid pBAD/HisA-1590. The constructed plasmid was confirmed by DNA sequencing. Then, pBAD/HisA-1590 was transferred into *E. coli* BL21. BL21/pBAD-HisA-*spd1590* was cultured in LB medium with 100 μg/ml Amp at 37°C in a shaking incubator. When the OD_600_ reached 0.6–0.8, 0.2% L-(+)-arabinose was added to the medium to induce the expression of SPD_1590, with continued incubation for 4–6 h. The cells were collected by centrifugation and washed three times with 1× PBS. After resuspending in PBS, the harvested cells were lysed by sonication. The His-SPD_1590 fusion protein was isolated by Ni-NTA, then digested with recombinant Enterokinase (Solarbio, China) to remove the His-tag. The purified protein was verified by 12% sodium dodecyl sulfate-polyacrylamide gel electrophoresis (SDS-PAGE) and mass spectrometry (LTQ Orbitrap XL, Thermo Fisher Scientific, United States) following the method of a previous study (Sun et al., 2011; Li et al., 2013). The purified SPD_1590 was used as an antigen for antibody preparation (Tianjin Sungene Biotech Co., China).

### Western Blot Analysis

Equivalent quantities of proteins were analyzed by 10–12% SDS-PAGE and then transferred onto polyvinylidene fluoride (PVDF) membranes (Millipore, United States), which were incubated with the anti-SPD_1590 antibody at 4°C. This was followed by incubation with horseradish peroxidase (HRP)-conjugated goat anti-mouse as a secondary antibody. The results were visualized using Clarity Western ECL Substrate (Bio-Rad, United States) and captured using ImageMaster 2D Platinum 6.0 (GE Healthcare, United States).

### Protein Preparation, iTRAQ Labeling, and Proteomics Analysis

Total proteins were extracted from WT and Δ*1590* strains cultivated in THY medium while in the exponential growth phase. A sample of 200 μg total protein was digested with trypsin (Promega, United States) at 37°C for 16 h, then lyophilized. An iTRAQ Reagent 8-plex kit (AB SCIEX, United States) was used to label peptide samples according to the manufacturer’s protocol. All samples were detected using an Orbitrap Fusion Lumos mass spectrometer (Thermo, United States). The parameters used for mass spectrometry have been described previously (Yang et al., 2016). Then, the acquired raw data were identified and quantified via Thermo Proteome Discovery Software 2.1.1.2.1. Quantitative analysis parameters were set as follows: Max. Missed Cleava: 2; Digestion mode: Trypsin; Variable modifications: Oxidation (M), Acetyl (Protein N-term), Phospho (STY); Static modifications: Carbamidomethyl (C), iTRAQ 8-plex.

Gene Ontology (GO) enrichment analysis was performed in Glue GO plug-in of Cytoscape (version 3.5.1) with *p* < 0.05 to analyze the biological processes of differently expressed proteins (Shannon et al., 2003).

### Adherence to and Invasion of A549 Lung Epithelial Cells by *S. pneumoniae* Strains

Adhesion and intracellular invasion capabilities were measured according to methods in a previous report (Yu et al., 2017). A549 cells were cultured in DMEM (Thermo Fisher Scientific, United States) with 10% fetal bovine serum (FBS; Thermo Fisher Scientific, United States) at 37°C in 5% CO_2_. Then, they were gently rinsed twice with PBS. *S. pneumoniae* strains were suspended in DMEM with 1% FBS and diluted to 1.0 × 10^7^ CFU/ml, while 1 ml dilution was added to each well of A549 cells and incubated for 2 h at 37°C.

For the adhesion assay, A549 cells were washed three times with 1× PBS to remove non-adherent bacteria and digested with 0.25% trypsin (Thermo Fisher Scientific, United States) for 2–3 min. Then, 800 μl cold 1× PBS with 0.025% Triton X-100 (pH 7.4) was added to dissolve A549 cells for 10 min. Next, 100 μl of each cell lysate was diluted with PBS and spread on a Columbia agar plate containing 5% sheep blood.

For the intracellular invasion assay, 15 μg/ml gentamicin (Gen) was added to the medium to kill the bacteria present in each A549 sample, and then the cells were washed with 1× PBS. Next, cells were digested with 0.25% trypsin and dissolved with 0.025% Triton X-100 in PBS. After dissolution, cell lysates were diluted with PBS, and 100 μl of each dilution was spread on a Columbia agar plate containing 5% sheep blood. All plates were cultured at 37°C, and bacterial colonies were counted after 24 h of culture. The adherence and intracellular invasion abilities were measured by determining the number of adherent or invasive bacteria. Each experiment was repeated in triplicate.

### Real-Time Quantitative PCR (RT-qPCR)

For *S. pneumoniae*, total RNA was extracted with TRIZOL (Invitrogen, United States) after pretreated with 50 mg/ml lysozyme (Sigma, United States). For A549, total RNA was directly extracted with TRIZOL according to the manufacturer’s instructions. The total RNA concentration was measured by NanoDrop 2000 spectrophotometer (Thermo Fisher Scientific, United States). The mRNA was reverse-transcribed using a HiScript II Q RT SuperMix for qPCR (+gDNA wiper) Kit (Vazyme, China). The qPCR reaction was performed with the AceQ qPCR kit (Vazyme, China). The expression of target genes was assayed using the StepOne System (Applied Biosystems, United States) and the primers listed in **Table 2** (CCL2-F to β-Actin-R or 16S rRNA-F to nanA-R). Actin or 16S rRNA was used as internal controls, and expression levels were analyzed by the comparative critical threshold (2^-ΔΔCT^) method (Xie et al., 2011; Livak and Schmittgen, 2012). All experiments were carried out in triplicate.

### Inductively Coupled Plasma Mass Spectrometry (ICP-MS) Analysis

Bacteria in the exponential growth phase (OD_600_ ∼0.6) were collected by centrifugation and washed three times with 1× PBS that had been pretreated with Chelex-100 resin. After drying with a Scanvac Freeze Dryer (Labgene Scientific, Switzerland), the dry weights were measured. Then, the dry cells were resuspended in 65% nitric acid and heated to 75°C for 20 min. After that, 1.8 ml ddH_2_O was added before centrifugation at 13,000 ×*g* for 5 min. The supernatants were collected and analyzed by ICP-MS (Optima 2000 DV, PerkinElmer, United States). All results were normalized to the dry weight of the cells. Each experiment was repeated in three independent replicates.

### Fluorescence Spectrometry

The binding of apo-1590 with hemin was detected with a fluorescence spectrometer (F7000, Hitachi, Japan). The parameters were set as follows: the excitation wavelength was 280 nm, the data were captured from 290 to 450 nm at room temperature, and the slit width of excitation was 2.5 nm and that of the emission beam was 10.0 nm. First, 1.2 mM hemin was added dropwise to 1.5 ml 2 μM apo-1590, and the fluorescent spectra were recorded until the fluorescence intensity was stable. Then, the titration curves were analyzed in Origin 8.0, and the binding affinity (Ka) was calculated with a Hill plot.

### Proteinase K Digestion Assay

Proteinase K was diluted with 25 mM Tris–HCl (containing 100 mM NaCl, pH = 7.4) to concentrations of 0, 2.5, 5, 7.5, and 10 μg/ml. Apo-1590 and 1590+hemin were dissolved in 50 mM Tris-HCl (containing 100 mM NaCl, pH = 7.4) to concentrations of 10 μg/ml. Then, 1 μl of diluted proteinase K was added to 9 μl apo-1590 or 1590+hemin to reach final proteinase K concentrations of 0, 0.25, 0.5, 0.75, and 1.0 μg/ml. All samples were heated to 37°C for 1 h. After that, the reaction was terminated by the addition of phenylmethanesulfonyl fluoride (PMSF) or the addition of 5× SDS loading buffer, followed by boiling for 5 min. The results were analyzed with 12% SDS-PAGE.

### Circular Dichroism Spectrometry

The secondary structure of apo-1590 and 1590+hemin (6 μM) in 25 mM Tris–HCl (pH = 7.4) was detected with circular dichroism (CD; Applied Photophysics, United Kingdom) spectrometry. The parameters used were derived from a previous study (Li et al., 2014). All captured results were calculated by CDPro and analyzed by Origin 8.0.

### Statistics

Data were statistically analyzed by GraphPad Prism 6.0 (GraphPad Software, United States). Differences between three groups were analyzed by two-tailed, unpaired Student’s *t*-tests, and data are expressed as mean ± standard (SD). Results were considered significant at *p* < 0.05.

## Results

### Homology Analysis of SPD_1590

**FIGURE 1:**
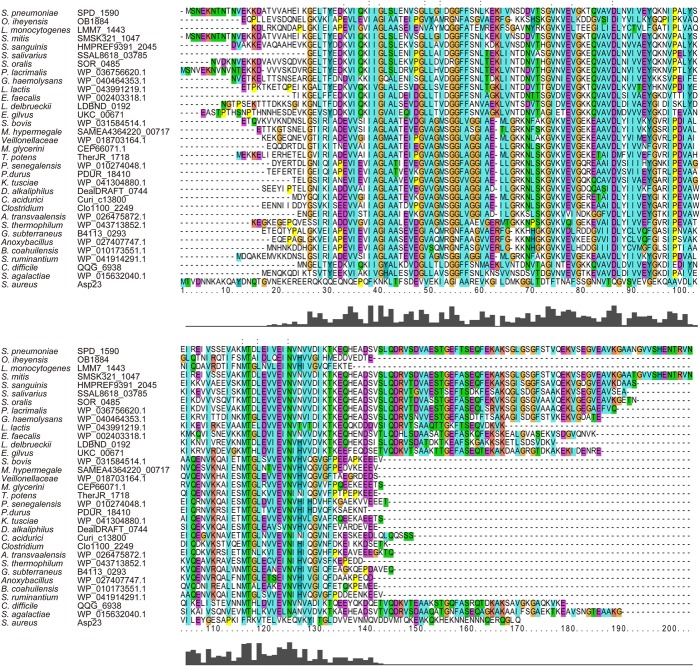
The multiple sequence alignment of SPD_1590 with homologous proteins in the various bacterial species.

Amino acid sequence alignment showed that SPD_1590 is highly conserved among Gram-positive bacteria (**Figure 1**). The region of SPD_1590 from amino acids 16–146 is completely identical to members of the alkaline shock protein (Asp23) family. In *Staphylococcus aureus*, Asp23 is a membrane-associated protein that is anchored to the membrane via the protein AmaP (Muller et al., 2014). The transcription of *asp23* is controlled by the alternative sigma factor σ^B^, which is involved in the response of the bacterium to environmental stress. As SPD_1590 has a high similarity to Asp23, we wondered whether SPD_1590 is also a membrane-associated protein.

Subsequently, we found that the homolog of SPD_1590 in *S. pneumoniae* R6 is SPR_1625, which was annotated as an Asp23/Gls24 family envelope stress response protein. Interestingly, a previous metal-affinity analysis indicated that SPR_1625 contained hemin-binding motifs and showed hemin-binding ability (Romero-Espejel et al., 2013). This initial evidence suggested that SPD_1590 may be involved in hemin transport in *Streptococcus* as a membrane-associated transporter.

### Deletion of *spd1590* Slows *S. pneumoniae* Growth and Affects Iron Uptake

**FIGURE 2:**
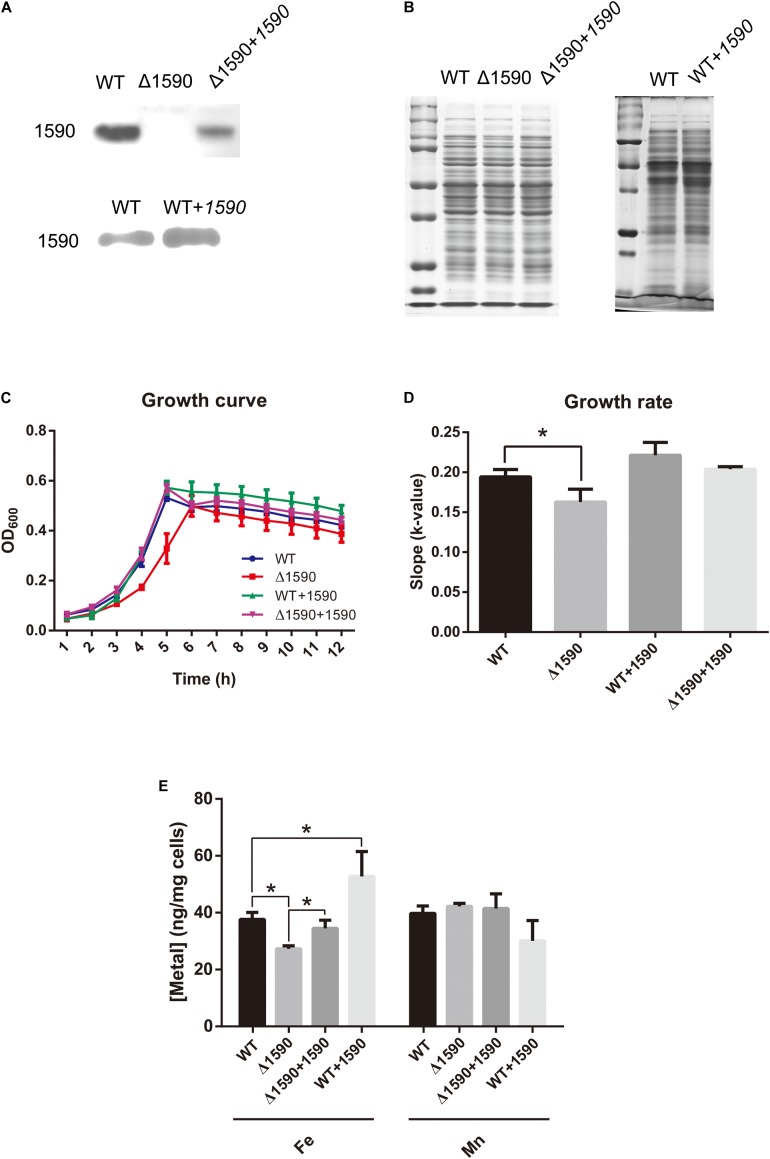
The deletion of *spd1590* affects bacterial growth and iron uptake. **(A)** The expression of SPD_1590 in WT, Δ*1590*, Δ*1590+1590*, and WT*+1590* strains as detected by western blotting. **(B)** SDS-PAGE of total proteins used as loading control for the western blotting experiments of **A**. The left one corresponds to the upper panel of **A** and the right one corresponds to the lower one. **(C)** The growth curves of WT, Δ*1590*, Δ*1590+1590*, and WT*+1590* strains cultured in C+Y medium. **(D)** The growth rates of WT and mutant D39 strains as per the growth curves in **C**. **(E)** The iron and manganese contents in WT, Δ*1590*, Δ*1590+1590*, and WT*+1590* strains as analyzed by ICP-MS (^∗^*p* < 0.05).

In order to ascertain the biological function of SPD_1590 *in vivo*, a deletion mutant (Δ*1590*) was obtained by homologous replacement, and an overexpressing strain (WT*+1590*) and complemented strain (Δ*1590+1590*) were constructed by transforming the plasmid pIB169-*spd1590* into WT and Δ*1590* D39. Western blotting showed that the mutant strains were successfully constructed (**Figures 2A,B**).

The growth curves of the WT and mutant D39 strains cultured on nutrition-restricted C+Y medium showed that deletion of *spd1590* slowed bacterial growth (**Figures 2C,D**). In contrast, Δ*1590+1590* showed a similar growth curve to WT D39, indicating that the complemented expression of 1590 recovered bacterial growth. However, overexpression of SPD_1590 did not increase the growth rate of D39, possibly indicating that SPD_1590 does not alter major metabolic pathways in *S. pneumoniae*. Notably, when cultured in THY, the growth of Δ*1590* is similar to that of WT D39.

To assess whether SPD_1590 is involved in iron uptake, the total metal content of strains WT D39, Δ*1590*, Δ*1590+1590*, and WT*+1590* was determined by ICP-MS. Compared with the WT strain, Δ*1590* had a lower iron content (*p* = 0.026) and WT*+1590* had a higher one (*p* = 0.044). The complemented expression of SPD_1590 in Δ*1590* restored the iron content to a level similar to that in WT D39. In contrast, there was no significant change in Mn content (**Figure 2E**).

### Expression of SPD_1590 Is Regulated by Hemin

**FIGURE 3:**
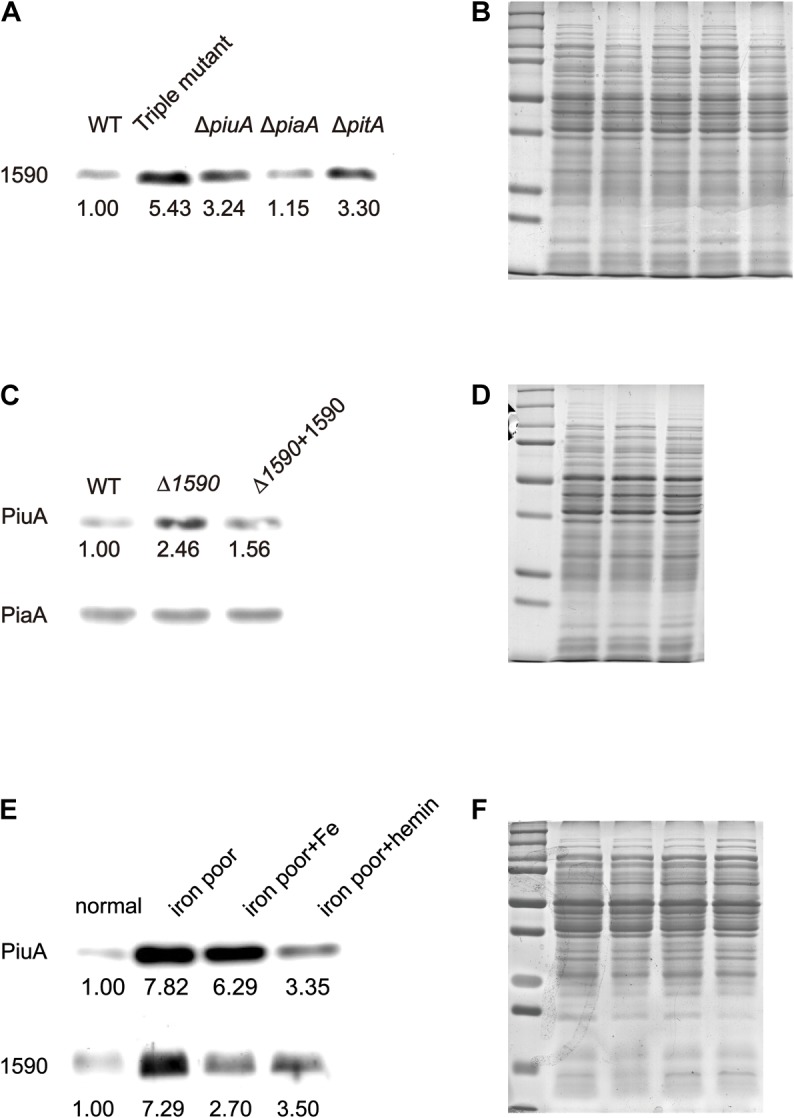
The expression of SPD_1590 is regulated by hemin. **(A)** The expression of SPD_1590 in WT, Triple mutant, Δ*pitA*, Δ*piaA*, and Δ*piuA* mutant D39 strains. **(C)** The expression of PiuA and PiaA in WT, Δ*1590*, and Δ*1590+1590* mutant D39 strains. **(E)** The expression of SPD_1590 and PiuA of WT D39 cultivated in normal, iron-poor, iron-poor+Fe, iron-poor+hemin media. **(B,D,F)** Total proteins were used as loading controls of the western blotting experiments as shown in **(A,C,E)**, respectively.

To better understand the role of SPD_1590 in iron uptake, its expression was detected via western blotting in the single mutants Δ*piuA*, Δ*piaA*, and Δ*pitA*, and in the triple mutant (Δ*piuA/*Δ*piaA/*Δ*pitA*), as these genes encode major iron transporters. SPD_1590 expression in the triple mutant was significantly upregulated, consistent with the previous iTRAQ results of our lab (Yang et al., 2016). In addition, SPD_1590 expression was also increased in the Δ*piuA* and Δ*pitA* strains (**Figures 3A,B**). PitA is a ferric ion transporter; however, there is hardly any free ferric ion in the host environment. In contrast, hemin and ferrichrome, transported by PiuA and PiaA, respectively, are the major iron sources for bacteria. Therefore, subsequent experiments focused on the relationship between SPD_1590 and PiuA and PiaA.

First, the expression levels of PiuA and PiaA were determined in the WT, Δ*1590*, and Δ*1590+1590* strains. Compared to the WT strain, the expression of PiuA was clearly upregulated in Δ*1590*, whereas it was similar to that in WT strain and the complemented Δ*1590+1590* strain (**Figures 3C,D**). In contrast, no significant differences were observed in the expression of PiaA in these three strains. Induction of PiuA, a major hemin transporter, in the absence of SPD_1590 suggests that SPD_1590 may also function as a hemin transporter. This potential function was further supported by the fact that, similar to PiuA, SPD_1590 expression was also induced during iron depletion and repressed in the presence of hemin (**Figures 3E,F**).

### Purified SPD_1590 Binds Hemin *in Vitro*

**FIGURE 4:**
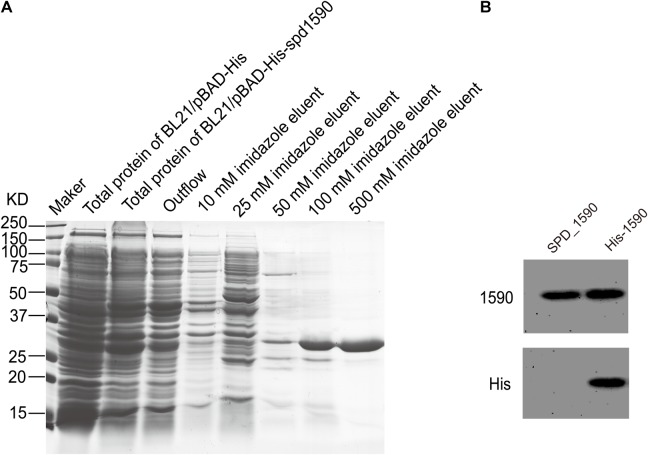
The purification of SPD_1590. **(A)** SPD_1590 was expressed with pBAD-His fusion system and purified by Ni-NTA column. **(B)** Western blotting assay for SPD_1590 expression using anti-SPD_1590 and anti-His6 antibody.

To further characterize the interaction between SPD_1590 and hemin, the *spd1590* gene was expressed in *E. coli* BL21 and purified by His-tag affinity chromatography. SDS-PAGE resulted in a single band of approximately 22 kDa, corresponding to the calculated molecular weight of SPD_1590 and indicating the purity of the purified protein (**Figure 4A**). Western blotting with anti-His and -1590 antibody confirmed the expression of SPD_1590 and the His-tag was successfully removed (**Figure 4B**).

**FIGURE 5:**
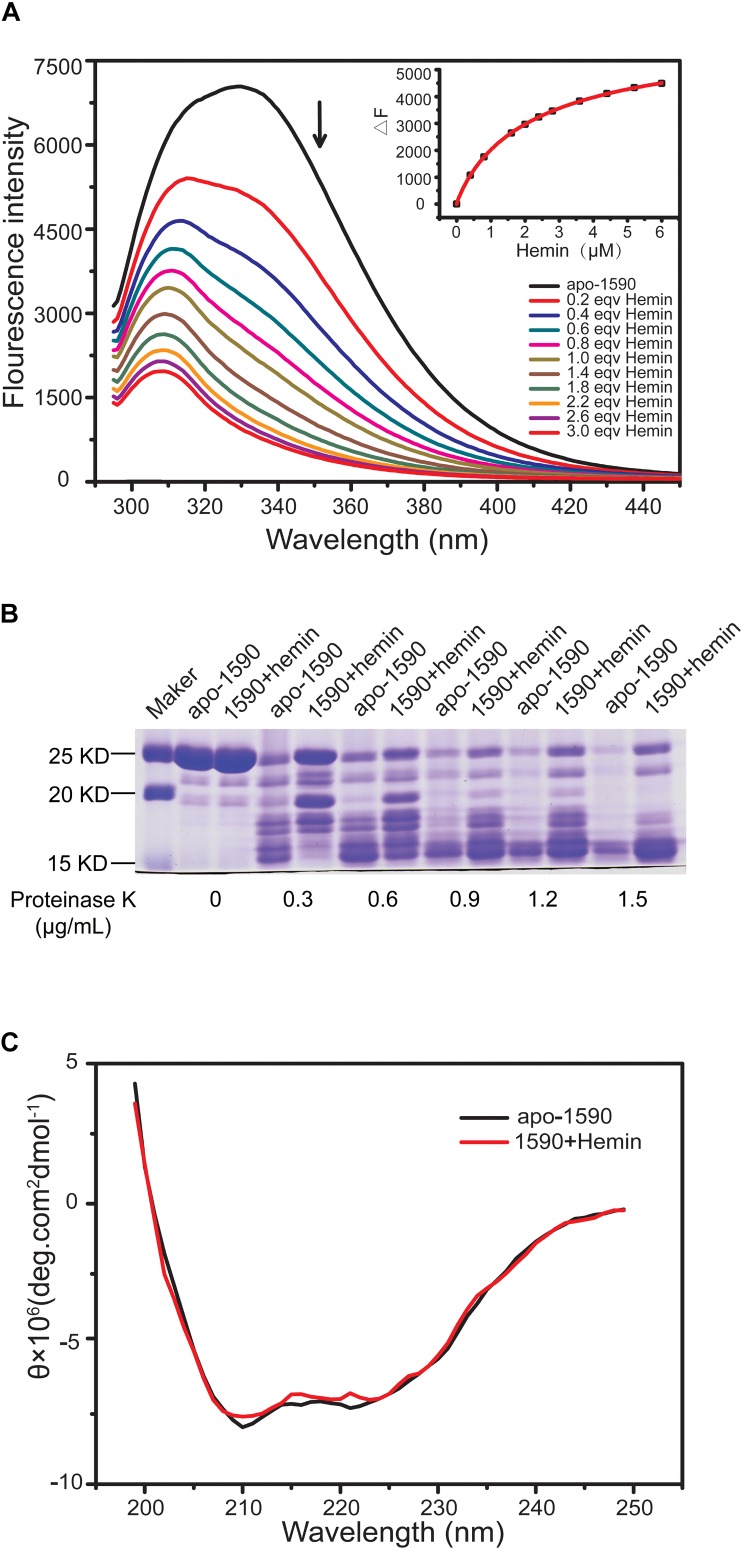
SPD_1590 binds with hemin. **(A)** Fluorescence spectra of hemin titration to apo-1590 (2 μM). **(B)** SDS-PAGE of apo-1590 and 1590+hemin pre-treated with 0, 0.3, 0.6, 0.9, 1.2, and 1.5 μg/ml proteinase K. **(C)** CD spectra of apo-1590 and 1590+hemin.

The binding of the purified SPD_1590 to hemin was measured by fluorescence quenching. The data were curve-fitted to the Hill plot, and the binding constant (Ka) of SPD_1590 to hemin was calculated to be 4.15 × 10^5^ M^-1^ (**Figure 5A**), which is similar to the Ka of PiuA to hemin determined with a similar method (Yao-Jin et al., 2018).

Due to the fact that binding to ligand tightens the binding pocket, the structure of the protein becomes more stable (Stodkilde et al., 2014; Cao et al., 2018). Next, the resistance of apo-SPD_1590 and hemin-bound SPD_1590 to proteinase digestion was measured using various concentrations of proteinase K (Kim et al., 2004). The results indicated that the metal-bound protein was more resistant to proteinase K degradation than apo-SPD_1590 protein under the same experimental conditions (**Figure 5B**). This effect was more evident at high concentrations of proteinase K. The high resistance ability of hemin-bound SPD_1590 to proteinase K cleavage suggested that hemin binding induced a more compacted protein structure.

To better understand the properties of SPD_1590, its secondary structure was determined by CD spectrometry. Approximately 49.87 ± 1.8% of the apo-protein was folded in α-helices, 27.67 ± 5.4% was in β-sheets, 15.17 ± 4.67% formed β-turns, and 14.4 ± 0.36% was devoid of symmetry. Hemin binding did not result in a significant change in the secondary structure of SPD_1590 (**Figure 5C** and **Table 3**). However, changes in the tertiary and quaternary structure of the protein upon hemin binding cannot be ruled out and might explain the differences in the protein properties observed, such as the increased resistance to proteinase degradation.

**Table 3:** Secondary structures of SPD_1590 with or without hemin binding.

Protein	α-Helix (%)	β-Sheet (%)	Turn (%)	Unordered (%)
SPD_1590	49.87 ± 1.80	27.67 ± 5.40	14.40 ± 0.36	15.17 ± 4.67
1590+hemin	47.57 ± 2.50	29.04 ± 3.50	13.50 ± 1.40	17.52 ± 1.60

### SPD_1590 Influences *S. pneumoniae* Pathogenicity and the Cell Inflammatory Response

**FIGURE 6:**
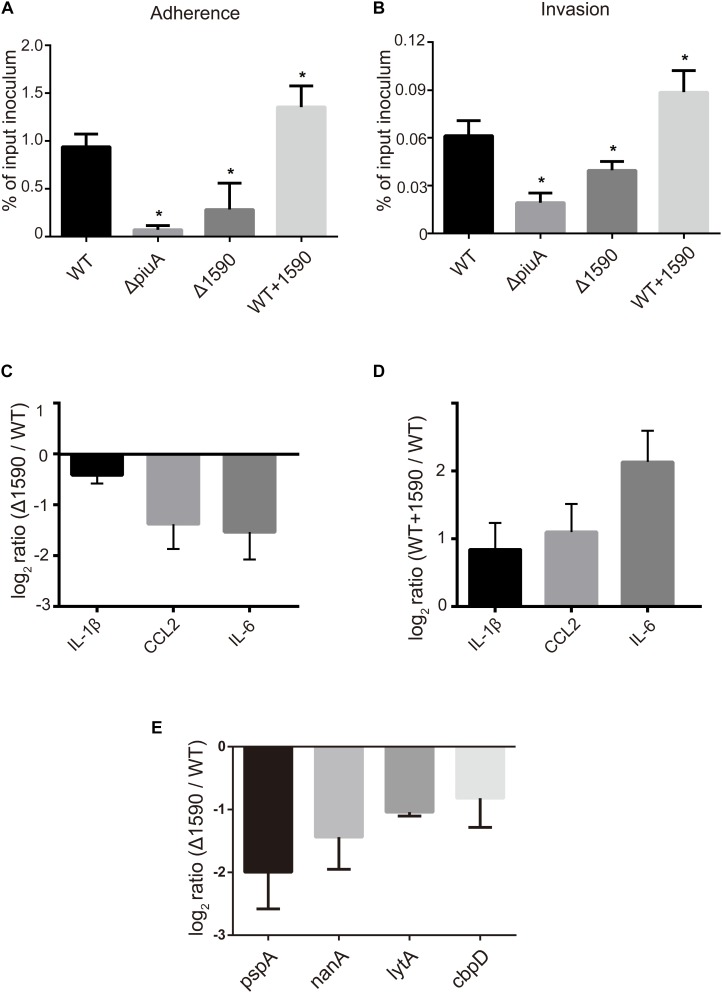
SPD_1590 promotes *S. pneumoniae* pathogenicity to A549 cells. **(A)** The adherence ability of WT, Δ*1590*, and WT*+1590* strains. **(B)** The intracellular invasion ability of WT, Δ*1590*, and WT*+1590* strains. ^∗^*p* < 0.05 compared to WT. **(C,D)** The mRNA expression levels of three inflammatory factors in A549 cells co-cultured with Δ*1590* and WT*+1590*, respectively, analyzed by RT-qPCR. Actin was used as an internal control. **(E)** The mRNA expression of four virulence factors in Δ*1590* compared to that in WT, analyzed by RT-qPCR. 16S rRNA was used as an internal control.

In addition to being involved in metal ion transport, many ABC transporters of pathogens, including PiuA, are also involved in adhesion to and invasion of host cells (Brown et al., 2001; Durmort and Brown, 2015; Nguyen and Gotz, 2016). Therefore, we investigated whether SPD_1590 was also important in *S. pneumoniae* infection of human lung carcinoma cells. To this end, the adherence and intracellular invasion of the WT, Δ*1590*, and WT*+1590*, as well as Δ*piuA* as a positive control, was measured in A549 cells. As shown in **Figures 6A,B**, *spd1590* deletion significantly reduced both the adhesion and intracellular invasion of *S. pneumoniae* compared to WT. In contrast, *spd1590* overexpression resulted in an increase in the number of bacteria adhering to and invading A549 cells. This result indicates that SPD_1590 is important for *S. pneumoniae* infection of human lung carcinoma cells.

To better understand the role of SPD_1590 during *S. pneumoniae* infection of human lung carcinoma cells, the expression of inflammatory factors was measured by RT-qPCR in A549 cells infected with the WT, Δ*1590*, and WT*+1590* strains. As shown in **Figures 6C,D**, compared with the WT strain, the expression of IL-1β, CCL2, and IL-6 was significantly reduced in cells infected with Δ*1590* and elevated in cells infected with WT*+1590*. This indicates that SPD_1590 alters the inflammatory response of A549 cells to *S. pneumoniae*.

### Deletion of *spd1590* Affects Several Metabolic Pathways and Bacterial Virulence Factors

Finally, we explored the role of SPD_1590 in the context of the bacterial metabolism by using iTRAQ-based proteomics to identify differentially expressed proteins between the WT and Δ*1590* strains. Using isotopic labels and three biological replicates, 1259 and 1275 proteins were detected in the WT and Δ*1590* strains, respectively. Relative quantitative analysis revealed 86 differentially expressed proteins (*p* < 0.05) in Δ*1590* compared with the WT, including 24 upregulated proteins (≥1.20-fold) and 62 downregulated proteins (≤0.83-fold). Detailed information on the differentially expressed proteins is provided in Supplementary Table S1.

The biological processes enriched in the 86 differentially expressed proteins were determined by GO analysis and included carbohydrate metabolism, transcription regulation, phosphate-containing compound metabolism, oxidation-reduction reactions, and nucleic acid phosphodiester bond hydrolysis (**Table 4**). Many proteins involved in carbohydrate metabolism, [Beta-galactosidase (putative), Beta-galactosidase 3, and Galactose-6-phosphate isomerase subunit LacB] and carbohydrate transport [five components of phosphotransferase (PTS) system] were downregulated, suggesting that Δ*1590* may have a low metabolic rate. This result is consistent with the fact that Δ*1590* showed a reduced growth rate compared to that of WT D39 in C+Y medium. In addition, four kinases including three histidine kinases regulating many important biological pathways and bacterial pathogenesis, were altered in Δ*1590*. Meanwhile, six proteins involved in oxidation-reduction process were changed indicating that cellular reactive oxygen species (ROS) altered by the deletion of *spd1590*. Additional proteins were notable even though they were not part of specific biological pathways. For instance, the expression levels of a cell wall surface anchor family protein (SPD_0335) and of the pneumococcal surface protein A (PspA) were 0.56- and 0.57-fold lower, respectively, in the mutant strain. These two proteins contribute to *S. pneumoniae* virulence and pathogenicity, suggesting that their reduced expression may be related to the attenuation of virulence in Δ*1590*.

**Table 4:** Summary of differently expressed proteins in Δ*1590* compared to WT D39.

Gene No.	Protein name	*Gene name*	Fold	*p*-Value
**Carbohydrate metabolic process**				
SPD_1971	Glycosyl hydrolase-related protein	*SPD_1971*	0.62	0.001
SPD_1974	Uncharacterized protein	*SPD_1974*	0.74	0.012
SPD_0562	Beta-galactosidase, putative	*bgaA*	0.73	0.000
SPD_0065	Beta-galactosidase 3 (EC 3.2.1.23)	*bgaC*	0.63	0.000
SPD_1634	Galactokinase (EC 2.7.1.6) (Galactose kinase)	*galK*	0.75	0.009
SPD_0071	Aldose 1-epimerase (EC 5.1.3.3) (Galactose mutarotase)	*galM*	0.79	0.047
SPD_1005	1,4-alpha-glucan branching enzyme GlgB (EC 2.4.1.18) (1,4-alpha-D-glucan:1,4-alpha-D-glucan 6-glucosyl-transferase) (Alpha-(1->4)-glucan branching enzyme) (Glycogen branching enzyme) (BE)	*glgB*	1.23	0.020
SPD_1052	Galactose-6-phosphate isomerase subunit LacB (EC 5.3.1.26)	*lacB*	0.65	0.001
SPD_0063	Beta-*N*-acetylhexosaminidase (EC 3.2.1.52)	*strH*	0.54	0.000
**Regulation of transcription, DNA-templated**				
SPD_0064	Transcriptional regulator, GntR family protein	*SPD_0064*	1.37	0.001
SPD_0158	DNA-binding response regulator	*SPD_0158*	1.27	0.012
SPD_1524	Transcriptional regulator, GntR family protein	*SPD_1524*	0.69	0.003
SPD_1605	Sugar-binding transcriptional regulator, LacI family protein	*SPD_1605*	0.76	0.006
SPD_1904	Arginine repressor	*argR*	0.75	0.023
SPD_0467	BlpS protein	*blpS*	0.66	0.011
SPD_2063	Response regulator	*comE*	0.77	0.001
SPD_1635	Galactose operon repressor	*galR*	0.78	0.002
**Carbohydrate transport**				
SPD_0066	PTS system, IIB component	*SPD_0066*	0.43	0.000
SPD_0068	PTS system, IID component	*SPD_0068*	0.45	0.007
SPD_0561	PTS system, IIC component, putative	*SPD_0561*	0.67	0.035
SPD_1047	PTS system, lactose-specific IIBC components	*lacE-2*	0.51	0.010
SPD_1048	PTS system, lactose-specific IIA component (EC 2.7.1)	*lacF-2*	0.73	0.002
**Phosphate-containing compound metabolic process**				
SPD_1165	Uncharacterized protein	*SPD_1165*	1.45	0.047
SPD_1445	Sensor histidine kinase (EC 2.7.3)	*SPD_1445*	0.71	0.010
SPD_1131	Carbamoyl-phosphate synthase large chain (EC 6.3.5.5) (Carbamoyl-phosphate synthetase ammonia chain)	*carB*	1.21	0.029
SPD_1557	Probable nicotinate-nucleotide adenylyltransferase (EC 2.7.7.18) [Deamido-NAD(+) diphosphorylase] [Deamido-NAD(+) pyrophosphorylase] (Nicotinate mononucleotide adenylyltransferase) (NaMN adenylyltransferase)	*nadD*	0.81	0.006
SPD_0745	Glycerol-3-phosphate acyltransferase (Acyl-PO4 G3P acyltransferase) (Acyl-phosphate–glycerol-3-phosphate acyltransferase) (G3P acyltransferase) (GPAT) (EC 2.3.1.n3) (Lysophosphatidic acid synthase) (LPA synthase)	*plsY*	1.29	0.008
SPD_1909	Sensor histidine kinase PnpS	*pnpS*	1.24	0.011
SPD_0632	Phosphomethylpyrimidine kinase (EC 2.7.4.7)	*thiD*	0.75	0.004
SPD_0525	Histidine kinase (EC 2.7.13.3)	*vncS*	0.77	0.004
**Oxidation-reduction process**				
SPD_1301	NADPH-dependent FMN reductase	*SPD_1301*	0.73	0.032
SPD_1985	Alcohol dehydrogenase, iron-containing (EC 1.1.1.1)	*SPD_1985*	0.75	0.000
SPD_0721	Bifunctional protein FolD [includes: Methylenetetrahydrofolate dehydrogenase (EC 1.5.1.5); Methenyltetrahydrofolate cyclohydrolase (EC 3.5.4.9)]	*folD*	1.22	0.022
SPD_0406	Ketol-acid reductoisomerase [NADP(+)] (KARI) (EC 1.1.1.86) (Acetohydroxy acid isomeroreductase) (AHIR) (Alpha-keto-beta-hydroxylacyl reductoisomerase) (Ketol-acid reductoisomerase type 1) (Ketol-acid reductoisomerase type I)	*ilvC*	0.68	0.001
SPD_0851	Dihydroorotate dehydrogenase B [NAD(+)], electron transfer subunit (Dihydroorotate oxidase B, electron transfer subunit)	*pyrK*	1.20	0.034
SPD_0636	Pyruvate oxidase (EC 1.2.3.3)	*spxB*	1.23	0.006
**Nucleic acid phosphodiester bond hydrolysis**				
SPD_0826	DNA polymerase III, delta prime subunit (EC 2.7.7.7)	*holB*	0.80	0.010
SPD_1105	Ribonuclease 3 (EC 3.1.26.3) (Ribonuclease III) (RNase III)	*rnc*	0.76	0.018
SPD_1020	Ribonuclease HII (RNase HII) (EC 3.1.26.4)	*rnhB*	0.82	0.016
**Other**				
SPD_0335	Cell wall surface anchor family protein	*SPD_0335*	0.56	0.000
SPD_0126	Pneumococcal surface protein A	*pspA*	0.57	0.000
SPD_1201	Phosphotransferase LicD3	*licD3*	0.82	0.010
SPD_1047	PTS system, lactose-specific IIBC components	*lacE-2*	0.51	0.010

*Fold represents fold changes of proteins in Δspd1590 vs. WT strain.*

To further explore this, the expression levels of several virulence factors were determined by RT-qPCR in D39 and Δ*1590*. Compared to levels in WT D39, levels of the virulence factors *pspA, nanA, lytA*, and *cbpD* were significantly reduced at mRNA level in Δ*1590* (**Figure 6E**). In addition, the decrease in *pspA* was consistent with the findings of the above mentioned proteomics analysis. As these virulence factors contribute to adherence, colonization, and migration, this result may reflect the mechanism by which *spd1590* expression influences *S. pneumoniae* pathogenicity in A549 cells and the cell inflammatory response.

## Discussion

Iron is an essential micronutrient for both eukaryotes and prokaryotes. However, it can also be toxic to organisms through the generation of ROS by Fenton chemistry and the Haber–Weiss reaction (Sheldon and Heinrichs, 2015). Thus, free bioavailable iron is rarely found in solution in biological systems, not only because of its insolubility but also as a mechanism for protecting the organism. Consequently, pathogens have evolved a multitude of complicated, and often redundant, mechanisms to acquire iron from the host (Cassat and Skaar, 2013), such as the ABC transporters PiaABC, PiuABC, and PitABC of *S. pneumonia* (Brown et al., 2001, 2002).

In addition to the above known iron transporters, this study aimed to clarify the role of SPD_1590 in iron uptake. Even though SPD_1590 was annotated as a general stress protein in the NCBI database, three initial indications suggested that it might be an iron transporter. First, its equivalent proteins, SPR_1625 in *S. pneumoniae* R6 was identified as a hemin binding protein. Second, it is upregulated at both the protein and mRNA levels in the Δ*piuA*/Δ*piaA*/Δ*pitA* triple mutant, in which iron uptake is deficient due to the absence of the three main iron transporters. Lastly, its homolog Asp23 is a membrane-associated protein, as the three known iron transporters. According to existing studies, proteins with hemin-binding capabilities may function as iron transporters, storage proteins, enzymes, or coenzyme (Benson and Rivera, 2013). Based on this information, we speculated that SPD_1590 was a potential iron transporter located on the cell surface that was responsible for iron uptake.

We showed that deletion of *spd1590* slowed *S. pneumoniae* growth in C+Y but not in THY medium. This result suggests that the three main ABC iron transporters are able to acquire sufficient iron for bacterial growth in the absence of SPD_1590 in a nutrient-rich environment. SPD_1590 is necessary for iron uptake only when the bacteria are cultured in an iron-limited environment. Thus, SPD_1590 may be a minor iron transporter, supplementing the three main transporters under iron-deficient conditions. Moreover, both metal content and protein expression analyses suggested that SPD_1590 was similar to PiuA, which is regulated by iron. Fluorescence spectroscopy analysis and measurement of the resistance of SPD_1590 to proteinase K treatment provided even stronger evidence that SPD_1590 has a similar affinity for hemin binding as PiuA. Piu (pneumococcal iron uptake) is one of the *S. pneumoniae* iron ABC transport systems and primarily transports hemin. PiuA is the lipoprotein component of the ABC transporter, located on the cell surface, which first binds hemin and then transports it to the membrane permease for subsequent passage through the cell membrane, with ATPase providing energy. Importantly, the Piu transport system is essential for *S. pneumoniae* virulence in the murine systemic and pulmonary models of infection. Therefore, we speculate that SPD_1590 is a minor hemin transporter and may contribute to bacterial virulence properties.

According to adherence, intracellular invasion, and virulence factor assays, SPD_1590 was found to play a role in the infection of host cells *in vitro*. Upon deletion of SPD_1590, the reduced virulence properties of bacteria induced a weak inflammatory reaction in host cells. In addition, iTRAQ-based proteomics analysis of Δ*1590* and the WT strain revealed several differentially expressed proteins involved in six biological processes. There were many proteins involved in carbohydrate metabolism and transport that were down-regulated in Δ*1590*. Especially, the decreased expression of Beta-galactosidase (putative), Beta-galactosidase 3, and Galactose-6-phosphate isomerase subunit LacB implied that the deletion of *spd1590* may affect TCA cycle and fatty acid metabolism, and thus reduce ATP production and suppress bacterial growth. It is interesting that several of these proteins are annotated as PTS system components, which respond to external or internal stimuli by inducing elaborate signal transduction pathways and are considered the bacterial “nervous system” (Aboulwafa and Saier, 2013). Meanwhile, six proteins involved in oxidation-reduction processes were changed in Δ*1590*. This suggests that SPD_1590 may have a role in the stress response, and we speculate that it is related to effect of the altered iron content on intracellular ROS by Fenton chemistry, leading to oxidative stress. More importantly, PspA, is an adhesin related to bacterial virulence, which blocks apolactoferrin (ALF)-mediated killing, allowing *S. pneumoniae* to escape this host defense mechanism (Shaper et al., 2004; Li-Korotky et al., 2010). This relies on PspA combining with lactoferrin to suppress complement activation (Ochs et al., 2008). Lactoferrin is an important participant and also an iron-binding protein in the host. The reduced expression of PspA was observed in both the proteomics and RT-qPCR analyses, the latter of which also demonstrated decreases in the expression of three additional virulence factors. NanA is a type of neuraminidase that promotes bacterial adherence, colonization, and migration (Manco et al., 2006). LytA participates in *S. pneumoniae* cell lysis to release other intracellular virulence factors (Bai et al., 2014). CbpD is believed to degrade the cell walls of target cells and can regulate iron-dependent biofilm formation (Eldholm et al., 2010). As expression of these virulence factors was reduced in the *spd1590* knockout, we believe that this is the reason for the impairments in host cell adherence and invasion in the Δ*1590* mutant, as well as the reduction in the inflammatory response of host cells in eliminating *S. pneumoniae*.

**FIGURE 7:**
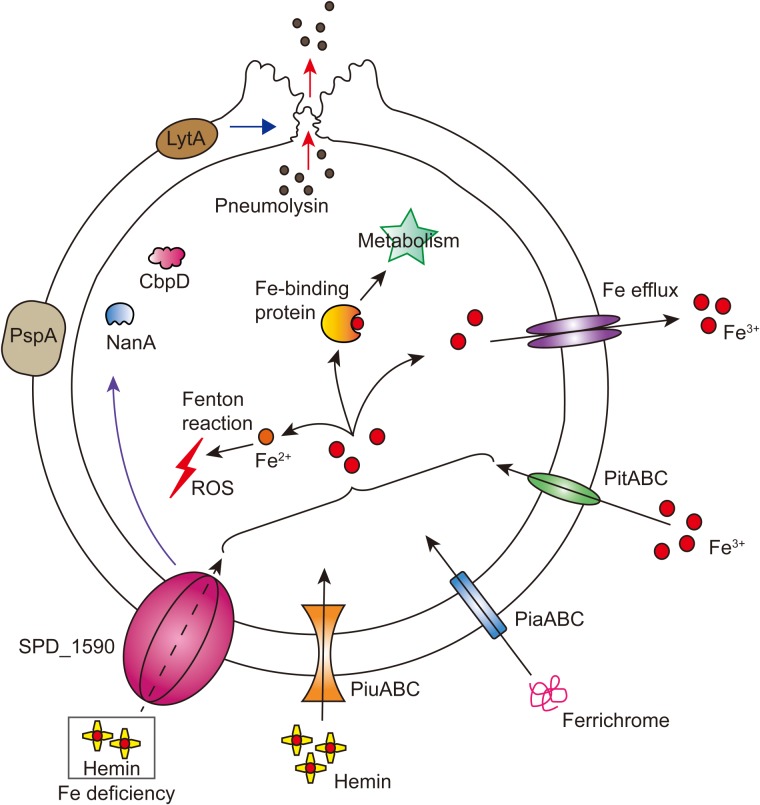
Integrated model of SPD_1590 function in *S. pneumoniae*. SPD_1590 works as a hemin transporter to supplement the function of PiuA. As a pleiotropic physiological protein, SPD_1590 can influence cellular ROS, carbohydrate metabolism and expression of several virulence factors, and thus monitors the pathogenicity of *S. pneumoniae* to host cells.

Overall, our results support the idea that SPD_1590 is a new class of iron transporter in *S. pneumoniae* that also contributes to bacterial virulence properties (**Figure 7**). Our results fill a gap in the previous understanding of the function of SPD_1590; rather than binding hemin as part of degradation or storage pathway, we have identified SPD_1590 is a type of hemin transporter. Further studies on the specific molecular mechanism used by SPD_1590 to acquire iron will help to gain insight into the iron-uptake strategies used by pathogenic bacteria.

## Data Availability

The mass spectrometry proteomics data have been deposited to the ProteomeXchange Consortium via the PRIDE (Vizcaino et al., 2016) partner repository with the dataset identifier PXD008814 (Username: reviewer85932@ebi.ac.uk, Password: fTJSYpSA).

## Author Contributions

XS, RG, and Q-YH designed the project and revised the paper. XM, JH, LZ, and XZ performed the experiment and data-analysis. XM and XS wrote the manuscript.

## Conflict of Interest Statement

The authors declare that the research was conducted in the absence of any commercial or financial relationships that could be construed as a potential conflict of interest.
