# Supplementary material for: A Novel Iron Transporter SPD_1590 in Streptococcus pneumoniae Contributing to Bacterial Virulence Properties
Source: Front Microbiol. 2018 Jul 20;9:1624. doi: 10.3389/fmicb.2018.01624 (PMC6062600; doi:10.3389/fmicb.2018.01624)
Supplement: Supplementary file 1 [file Table_1.DOCX]

Table S1. The complete list of differently expressed proteins

| **Entry** | **Protein names** | **Gene name** | **Gene NO.** | **Fold** | **p-value** |
| --- | --- | --- | --- | --- | --- |
| Q04KG8 | 1,4-alpha-glucan branching enzyme GlgB (EC 2.4.1.18) (1,4-alpha-D-glucan:1,4-alpha-D-glucan 6-glucosyl-transferase) (Alpha-(1->4)-glucan branching enzyme) (Glycogen branching enzyme) (BE) | glgB | SPD_1005 | 1.23 | 0.020 |
| Q04MM6 | 50S ribosomal protein L14 | rplN | SPD_0203 | 1.26 | 0.036 |
| Q04MR3 | 6,7-dimethyl-8-ribityllumazine synthase (DMRL synthase) (LS) (Lumazine synthase) (EC 2.5.1.78) | ribH | SPD_0166 | 0.77 | 0.018 |
| A0A0H2ZPZ8 | 6-phospho-beta-galactosidase (EC 3.2.1.85) (Beta-D-phosphogalactoside galactohydrolase) (PGALase) (P-beta-Gal) (PBG) | lacG-2 | lacG | 0.73 | 0.012 |
| A0A0H2ZMS1 | ABC transporter ATP-binding protein | SPD_0687 | SPD_0687 | 0.78 | 0.003 |
| A0A0H2ZRU3 | ABC transporter, ATP-binding protein | SPD_1137 | SPD_1137 | 1.47 | 0.006 |
| A0A0H2ZQK8 | ABC transporter, substrate-binding protein | SPD_0090 | SPD_0090 | 0.49 | 0.001 |
| A0A0H2ZM28 | ABC transporter, transmembrane protein Vexp1 | vex1 | SPD_0521 | 0.76 | 0.028 |
| A0A0H2ZMY2 | Acetyltransferase, GNAT family protein | SPD_0905 | SPD_0905 | 1.23 | 0.010 |
| A0A0H2ZLH7 | Alcohol dehydrogenase, iron-containing (EC 1.1.1.1) | SPD_1985 | SPD_1985 | 0.75 | 0.000 |
| A0A0H2ZRU4 | Aldose 1-epimerase (EC 5.1.3.3) (Galactose mutarotase) | galM | SPD_0071 | 0.79 | 0.047 |
| A0A0H2ZN43 | Amino acid ABC transporter, permease protein | SPD_1290 | SPD_1290 | 1.25 | 0.026 |
| A0A0H2ZPQ9 | Arginine repressor | argR | SPD_1904 | 0.75 | 0.023 |
| A0A0H2ZQE5 | Beta-galactosidase 3 (EC 3.2.1.23) | bgaC | SPD_0065 | 0.63 | 0.000 |
| A0A0H2ZMI1 | Beta-galactosidase, putative | bgaA | SPD_0562 | 0.73 | 0.000 |
| A0A0H2ZQY4 | Beta-N-acetylhexosaminidase (EC 3.2.1.52) | strH | SPD_0063 | 0.54 | 0.000 |
| Q04L84 | Bifunctional protein FolD [Includes: Methylenetetrahydrofolate dehydrogenase (EC 1.5.1.5); Methenyltetrahydrofolate cyclohydrolase (EC 3.5.4.9)] | folD | SPD_0721 | 1.22 | 0.022 |
| A0A0H2ZP79 | BlpS protein | blpS | SPD_0467 | 0.66 | 0.011 |
| Q04K48 | Carbamoyl-phosphate synthase large chain (EC 6.3.5.5) (Carbamoyl-phosphate synthetase ammonia chain) | carB | SPD_1131 | 1.21 | 0.029 |
| A0A0H2ZQA7 | Cation efflux family protein | SPD_1384 | SPD_1384 | 0.75 | 0.009 |
| A0A0H2ZP65 | Cell division protein FtsL | ftsL | SPD_0305 | 0.77 | 0.000 |
| A0A0H2ZM17 | Cell wall surface anchor family protein | SPD_0335 | SPD_0335 | 0.56 | 0.000 |
| A0A0H2ZND3 | Choline binding protein F | cbpF | SPD_0357 | 0.69 | 0.028 |
| A0A0H2ZNR9 | Cof family protein | SPD_0816 | SPD_0816 | 0.76 | 0.028 |
| A0A0H2ZQI3 | Cof family protein | SPD_1280 | SPD_1280 | 1.28 | 0.045 |
| A0A0H2ZQ41 | Competence-stimulating peptide type 1 | comC1 | SPD_2065 | 0.41 | 0.003 |
| A0A0H2ZMC4 | Dihydroorotate dehydrogenase B (NAD(+)), electron transfer subunit (Dihydroorotate oxidase B, electron transfer subunit) | pyrK | SPD_0851 | 1.20 | 0.034 |
| A0A0H2ZM57 | DNA polymerase III, delta prime subunit (EC 2.7.7.7) | holB | SPD_0826 | 0.80 | 0.010 |
| A0A0H2ZQE7 | DNA-binding response regulator | SPD_0158 | SPD_0158 | 1.27 | 0.012 |
| A0A0H2ZR12 | Endo-beta-N-acetylglucosaminidase, putative | SPD_0444 | SPD_0444 | 0.69 | 0.000 |
| A0A0H2ZNM7 | Galactokinase (EC 2.7.1.6) (Galactose kinase) | galK | SPD_1634 | 0.75 | 0.009 |
| A0A0H2ZLH0 | Galactose operon repressor | galR | SPD_1635 | 0.78 | 0.002 |
| Q04KC2 | Galactose-6-phosphate isomerase subunit LacB (EC 5.3.1.26) | lacB | SPD_1052 | 0.65 | 0.001 |
| A0A0H2ZNN3 | General stress protein 24, putative | SPD_1590 | SPD_1590 | 0.49 | 0.000 |
| Q04L63 | Glycerol-3-phosphate acyltransferase (Acyl-PO4 G3P acyltransferase) (Acyl-phosphate--glycerol-3-phosphate acyltransferase) (G3P acyltransferase) (GPAT) (EC 2.3.1.n3) (Lysophosphatidic acid synthase) (LPA synthase) | plsY | SPD_0745 | 1.29 | 0.008 |
| A0A0H2ZNP7 | Glycosyl hydrolase-related protein | SPD_1971 | SPD_1971 | 0.62 | 0.001 |
| A0A0H2ZKZ7 | Histidine kinase (EC 2.7.13.3) | vncS | SPD_0525 | 0.77 | 0.004 |
| A0A0H2ZPH9 | Integral membrane protein | SPD_0988 | SPD_0988 | 1.25 | 0.047 |
| A0A0H2ZLQ5 | Iron-compound ABC transporter, ATP-binding protein | SPD_0918 | SPD_0918 | 0.76 | 0.005 |
| Q04M32 | Ketol-acid reductoisomerase (NADP(+)) (KARI) (EC 1.1.1.86) (Acetohydroxy-acid isomeroreductase) (AHIR) (Alpha-keto-beta-hydroxylacyl reductoisomerase) (Ketol-acid reductoisomerase type 1) (Ketol-acid reductoisomerase type I) | ilvC | SPD_0406 | 0.68 | 0.001 |
| A0A0H2ZMT9 | Lipoprotein, putative | SPD_0184 | SPD_0184 | 1.50 | 0.035 |
| A0A0H2ZP54 | NADPH-dependent FMN reductase | SPD_1301 | SPD_1301 | 0.73 | 0.032 |
| A0A0H2ZM90 | Oligopeptide ABC transporter, pemease protein | SPD_1168 | SPD_1168 | 1.26 | 0.015 |
| A0A0H2ZP09 | Phosphomethylpyrimidine kinase (EC 2.7.4.7) | thiD | SPD_0632 | 0.75 | 0.004 |
| A0A0H2ZNH6 | Phosphopantothenoylcysteine decarboxylase (EC 4.1.1.36) | coaC | SPD_1089 | 0.79 | 0.050 |
| A0A0H2ZNB4 | Phosphotransferase LicD3 | licD3 | SPD_1201 | 0.82 | 0.010 |
| A0A0H2ZMX6 | Pneumococcal surface protein A | pspA | SPD_0126 | 0.57 | 0.000 |
| A0A0H2ZM20 | Probable nicotinate-nucleotide adenylyltransferase (EC 2.7.7.18) (Deamido-NAD(+) diphosphorylase) (Deamido-NAD(+) pyrophosphorylase) (Nicotinate mononucleotide adenylyltransferase) (NaMN adenylyltransferase) | nadD | SPD_1557 | 0.81 | 0.006 |
| A0A0H2ZMS0 | PTS system, IIB component | SPD_0066 | SPD_0066 | 0.43 | 0.000 |
| A0A0H2ZQC7 | PTS system, IIC component, putative | SPD_0561 | SPD_0561 | 0.67 | 0.035 |
| A0A0H2ZKY1 | PTS system, IID component | SPD_0068 | SPD_0068 | 0.45 | 0.007 |
| A0A0H2ZP74 | PTS system, lactose-specific IIA component (EC 2.7.1.-) | lacF-2 | SPD_1048 | 0.73 | 0.002 |
| A0A0H2ZLK0 | PTS system, lactose-specific IIBC components | lacE-2 | SPD_1047 | 0.51 | 0.010 |
| A0A0H2ZP58 | Pyruvate oxidase (EC 1.2.3.3) | spxB | SPD_0636 | 1.23 | 0.006 |
| A0A0H2ZLR3 | Response regulator | comE | SPD_2063 | 0.77 | 0.001 |
| Q04K72 | Ribonuclease 3 (EC 3.1.26.3) (Ribonuclease III) (RNase III) | rnc | SPD_1105 | 0.76 | 0.018 |
| Q04KF4 | Ribonuclease HII (RNase HII) (EC 3.1.26.4) | rnhB | SPD_1020 | 0.82 | 0.016 |
| A0A0H2ZQU9 | Sensor histidine kinase (EC 2.7.3.-) | SPD_1445 | SPD_1445 | 0.71 | 0.010 |
| A0A0H2ZML6 | Sensor histidine kinase PnpS | pnpS | SPD_1909 | 1.24 | 0.011 |
| A0A0H2ZMS8 | Sugar isomerase domain protein AgaS (EC 5.-.-.-) | agaS | SPD_0070 | 0.70 | 0.000 |
| A0A0H2ZPF9 | Sugar-binding transcriptional regulator, LacI family protein | SPD_1605 | SPD_1605 | 0.76 | 0.006 |
| A0A0H2ZLC3 | Transcriptional regulator ComX1 (Transcriptional regulator ComX2) | comX1 | comX2 | 0.73 | 0.015 |
| A0A0H2ZQS8 | Transcriptional regulator, GntR family protein | SPD_1524 | SPD_1524 | 0.69 | 0.003 |
| A0A0H2ZMA3 | Transcriptional regulator, GntR family protein | SPD_0064 | SPD_0064 | 1.37 | 0.001 |
| A0A0H2ZKZ2 | Transketolase N-terminal subunit | SPD_1958 | SPD_1958 | 0.81 | 0.045 |
| A0A0H2ZMF4 | Type I restriction-modification system, S subunit, putative | SPD_0451 | SPD_0451 | 1.25 | 0.047 |
| A0A0H2ZMH3 | Uncharacterized protein | SPD_1591 | SPD_1591 | 0.32 | 0.000 |
| A0A0H2ZND0 | Uncharacterized protein | SPD_0568 | SPD_0568 | 0.42 | 0.011 |
| A0A0H2ZMQ5 | Uncharacterized protein | SPD_1588 | SPD_1588 | 0.59 | 0.011 |
| A0A0H2ZPL4 | Uncharacterized protein | SPD_0634 | SPD_0634 | 0.68 | 0.044 |
| A0A0H2ZND4 | Uncharacterized protein | SPD_0466 | SPD_0466 | 0.70 | 0.001 |
| A0A0H2ZMX2 | Uncharacterized protein | SPD_0582 | SPD_0582 | 0.74 | 0.012 |
| A0A0H2ZR86 | Uncharacterized protein | SPD_1974 | SPD_1974 | 0.74 | 0.012 |
| A0A0H2ZMP5 | Uncharacterized protein | SPD_1928 | SPD_1928 | 0.78 | 0.029 |
| A0A0H2ZMR0 | Uncharacterized protein | SPD_0488 | SPD_0488 | 0.81 | 0.045 |
| A0A0H2ZP61 | Uncharacterized protein | SPD_0517 | SPD_0517 | 0.82 | 0.006 |
| A0A0H2ZPR7 | Uncharacterized protein | SPD_1294 | SPD_1294 | 0.83 | 0.000 |
| A0A0H2ZLP2 | Uncharacterized protein | SPD_1522 | SPD_1522 | 1.20 | 0.015 |
| A0A0H2ZMT6 | Uncharacterized protein | SPD_1166 | SPD_1166 | 1.21 | 0.013 |
| A0A0H2ZQ32 | Uncharacterized protein | SPD_1277 | SPD_1277 | 1.39 | 0.028 |
| A0A0H2ZM35 | Uncharacterized protein | SPD_1165 | SPD_1165 | 1.45 | 0.047 |
| O54519 | Uncharacterized protein SPD_2303 | orf3 | SPD_2303 | 0.43 | 0.040 |
| A0A0H2ZLK6 | Universal stress protein | SPD_1793 | SPD_1793 | 1.32 | 0.030 |
| Q04LF3 | UPF0298 protein SPD_0651 | SPD_0651 | SPD_0651 | 0.50 | 0.032 |
| A0A0H2ZMV8 | Uracil-xanthine permease | uraA | SPD_1141 | 1.22 | 0.022 |
| Q04L26 | Xaa-Pro dipeptidyl-peptidase (EC 3.4.14.11) (X-Pro dipeptidyl-peptidase) (X-prolyl-dipeptidyl aminopeptidase) (X-PDAP) | pepX | SPD_0787 | 0.78 | 0.034 |

*Fold represents fold changes of proteins in △spd1590 and WT strain.*
